# Human adipose-derived stem cells inhibit bioactivity of keloid fibroblasts

**DOI:** 10.1186/s13287-018-0786-4

**Published:** 2018-02-21

**Authors:** Xiuxia Wang, Yan Ma, Zhen Gao, Jun Yang

**Affiliations:** 1 Department of Plastic and Reconstructive Surgery, Shanghai 9th People’s Hospital, Shanghai Jiao Tong University School of Medicine, 639 Zhi Zao Ju Road, Shanghai, 200011 China; 2 Division of Plastic Surgery, Xinjiang Korla Bazhou People’s Hospital, Xinjiang, China

**Keywords:** Adipose-derived stem cells, Keloid fibroblasts, Conditioned medium

## Abstract

**Background:**

A keloid is a fibroproliferative disorder occurring in wounds characterized by an exaggerated response to injury. To date, no effective cure has been identified. As multipotent stem cells, human adipose-derived stem cells (ADSCs) may show the possibility for curing diseases such as fibrosis. This study sought to explore the potential role of human ADSCs in curing keloids.

**Methods:**

After culture in conditioned medium, gene and protein expression of keloid fibroblasts was examined using real-time polymerase chain reaction (RT-PCR) and Western blotting, while analysis of the cell cycle was used to measure the proliferative properties of the cells. Furthermore, ex vivo explant cultures were used to test the effects of ADSC-conditioned medium (ADSC-CM) on CD31^+^ and CD34^+^ expression in keloid tissue.

**Results:**

Our experimental results show that ADSC-CM was able to attenuate extracellular matrix-related gene expression as well as decrease protein expression. Cell proliferation was significantly suppressed in our study. CD31^+^ and CD34^+^ vessels in ex vivo explants were reduced by 55% and 57% in treatment groups compared with control groups.

**Conclusions:**

Human ADSC-CM significantly inhibited keloid fibroblast-related bioactivities.

## Background

A keloid caused by any traumatic stimulus is characterized by hyperproliferation of fibroblasts, excessive deposition of extracellular matrix (ECM), including collagen 1 (COL1) and collagen 3 (COL3), and constant invasion of normal tissue [1]. In the clinic, many methods including surgery, triamcinolone injection, and radical therapy have been used to treat keloids, but none of them have proven to be efficacious therapies and they may also give rise to new discomforts, causing discontent and complaints among patients. Better knowledge of the molecular mechanisms underlying the development of keloids would help the development of an effective therapeutic method.

Over recent decades, scientists have found that although fibroblasts isolated from keloid tissue have the same spindle-shaped morphology as normal fibroblasts, their gene expression and biological behavior are different. Keloid fibroblasts (KFs) produce more ECM, proliferate faster, and are more invasive than normal fibroblasts [2]. According to published literature, the expression levels of biologically active isoforms of transforming growth factor (TGF)-β ligands and their receptors are markedly elevated in KFs. Furthermore, TGF-β plays an important role in cell proliferation and collagen synthesis in KFs, and KFs display a distinct sensitivity to TGF-β stimulation [3]. In addition to the TGF-β signaling pathway, a number of other cytokines have been reported to be dysregulated in keloid pathogenesis and recurrence, such as insulin-like growth factor (IGF)-1 [4] and vascular endothelial growth factor (VEGF) [5]. All these secreted molecules regulate every aspect of keloid occurrence and development, including ECM production and deposition, cell proliferation, inflammation, immunoreaction, and angiogenesis. Therefore, a method which could suppress cytokine secretion or block signal transduction widely may be a good choice for curing keloids.

Since mesenchymal stromal cells (MSCs) were first identified in 1970 [6], and a detailed description of their trilineage potential was revealed [7], knowledge of the potential function of these cells has vastly increased. MSCs from adipose tissue may be easier to obtain and expand rapidly in vitro to generate an effective dose. As has been documented, human adipose-derived stem cells (ADSCs) have the same properties as MSCs from bone marrow [8, 9], and are currently being considered as potential therapeutic strategies for a number of diseases.

## Methods

### ADSC isolation and cultivation

Human adipose tissue samples were obtained from patients who had undergone lipoplasty. After rinsing three times with phosphate-buffered saline (PBS), the adipose tissue samples were digested with 0.1% collagenase IV (Roche Diagnostic, Mannheim, Germany) for 1 h. The suspension was then centrifuged to obtain the adipose-deprived stem cells. ADSCs were cultured in Dulbecco’s modified Eagle’s medium (DMEM) with 10% fetal bovine serum (FBS), 100 U/mL penicillin, and 100 mg/mL streptomycin (Gibco, Invitrogen, Carlsbad, CA, USA) at 37 °C in 5% CO_2_. After the ADSCs reached 80–90% confluence, the culture medium was changed to DMEM/F12 and then, 24 h later, the medium was collected, processed by centrifugation at 300 × *g* for 5 min and filtered with a 0.22-μm syringe filter (Jet Bio-Filtration, Guangzhou, China) and stored at −80 °C. Cells at passage 2–6 were used in this experiment. All experiments are approved by Shanghai Jiao Tong University of Medicine ethics committee and all the patients had provided informed content.

### Keloid scar cell isolation and culture

Twelve keloids from five men and three women were obtained after excision from the corresponding sites. Samples were soaked in chloromycetin for 30 min, cut into pieces as small as possible, and then digested with 0.2% collagenase IV for 4 h at 37 °C. After centrifugation, cells were suspended in DMEM with 10% FBS, 100 U/mL penicillin, and 100 mg/mL streptomycin at 37 °C in 5% CO_2_. Cells at passage 2–3 were used in this experiment. All these experiments were approved by Shanghai Jiao Tong University of Medicine ethics committee and all patients had provided informed content.

### Flow cytometric analysis

Human ADSCs (passage 3) were harvested and washed three times in PBS. The cell suspension was incubated with fluorescein isothiocyanate (FITC)-conjugated antibodies against CD29, CD44, CD45, CD90, CD105, CD31, and CD34 (Santa Cruz Biotechnology, Inc., Santa Cruz, CA, USA) at 37 °C for 30 min in the dark, washed, and resuspended in PBS and detected by flow cytometry (BD Biosciences, San Jose, CA, USA).

### Adipogenic and osteogenic differentiation

Human ADSCs at passages 3–5 were seeded into six-well plates that were pre-coated with a 0.1% gelatin solution (Cyagen Bioscience, Inc., Guangzhou, China) at a density of 10^5^ cells per well and allowed to reach 80–90% confluence. Adipogenic differentiation was induced using a basic medium with 0.5 μmol/L dexamethasone, 0.5 mmol/L 3-isobutyl-1-methylxanthine, 0.1 mmol/L rosiglitazone, and 100 IU insulin for 2 weeks (Cyagen Bioscience, Inc., HUXMD-90031). Osteogenic differentiation was achieved by incubating the cells in basic medium containing 0.1 μmol/L dexamethasone, 50 μmol/L ascorbic acid, and 10 mmol/L β-glycerophosphate for 3 weeks (Cyagen Bioscience, Inc., HUXMD-90021). Medium was replaced every 3 days.

At the endpoint, cells were fixed with 4% paraformaldehyde in PBS for 15 min at room temperature and stained with specific Oil Red O and Alizarin Red S following the manufacturers’ instructions to assess adipogenic and osteogenic differentiation, respectively. Stained ADSCs were counted under a light microscope (Olympus, Tokyo, Japan).

### Indirect coculture

To prepare conditioned medium (CM), human ADSCs from six individuals were cultured in DMEM/F12 for 24 h. The medium was centrifuged and the supernatant was removed without disturbing the pellet of cell debris. The conditioned medium was stored at −80 °C until use.

### Quantitative real-time polymerase chain reaction (qRT-PCR)

qRT-PCR was performed as previously reported [10]. Briefly, total RNA was extracted from KFs after 5 days of culture with ADSC-CM using an RNA isolation kit (Takara Bio, Shiga, Japan). RNA purity was evaluated by calculating the A260/A280 ratio between values of 1.8 and 2.0. The primer pairs used for gene amplification were as follows: TGF-beta1: forward AAGGACCTCGGCTGGAAGTG, reverse CCGGGTTATGCTGGTTGTA; COL1: forward GGCGGCCAGGGCTCCGACCC, reverse AATTCCTGGTCTGGGGCACC; COL3: forward TGGTGTTGGAGCCGCTGCCA, reverse CTCAGCACTAGAATCTGTCC; MMP1: forward GGAGCTGTAGATGTCCTTGGGGT, reverse GCCACAACTGCCAAATGGGCTT; MMP3: forward AGGACAAAGCAGGATCACAGTTG, reverse CCTGGTACCCACGGAACCT; GAPDH: forward TCACCATCTTCCAGGAGCG, reverse CTGCTTCACCACCTTCTTGA. The results from three independent reactions were used to determine relative gene expression, which was normalized against the expression level of GAPDH.

### Cell cycle

KFs were collected after culture with ADSC-CM for 24 h, rinsed once with PBS, and fixed with 70% alcohol overnight. Subsequent steps were performed according to the instructions supplied with the Cell Cycle Kit (Qihai Biotechnology, Shanghai, China), and flow cytometric analyses were performed using a flow cytometer (Beckman Coulter) equipped with ModiFit LT v2.0 software.

### Cell invasion assay

Once the KFs reached 90% confluence, they were collected and resuspended at 1 × 10^5^/mL in 0.5 mL ADSC-CM and the cell suspension was added to the upper chambers of 24-well transwell® plates (Merck Millipore, Darmstadt, Germany) with 1 mL DMEM containing 10% FBS placed in the lower chamber. After culturing for 24 h, the upper chamber cells were stabbed with cotton swab and the chamber were stained with DAPI, and then counted in five random fields.

### Ex vivo explant culture of human keloid tissue

After harvesting excised keloid tissue under aseptic conditions, the epidermis was removed, and the remaining dermis was cut into 3 × 2 × 2 mm pieces using a scalpel. The dermal fragments were divided into two groups, seeded into 6-cm culture dishes, and cultured for 3 days in 3 mL of DMEM containing 10% FBS as described previously [11]. After tissue attachment, the medium was replaced with fresh medium or conditioned medium. After culture for 8 days, the explants were collected.

### Histological and immunohistochemical analyses

The keloid explant specimens were fixed in 4% paraformaldehyde at 4 °C overnight, embedded in paraffin blocks, and sectioned at 5-μm thickness. The sections were then stained with hematoxylin and eosin (H&E) for routine examination. In addition, the keloid sections were incubated with antibodies against CD31 and CD34 at a dilution of 1:500. The bound antibodies were visualized using 3,3′-diaminobenzidine (DAB) as a chromogen (Dako, Glostrup, Denmark), and the slides were counterstained with hematoxylin. The numbers of CD31^+^ and CD34^+^ vessels were evaluated in six randomly selected fields under the microscope.

### Western blotting analysis

After 24 or 48 h of coculture, total protein was extracted with RIPA lysis buffer as described previously [12]. The protein bands were visualized using an enhanced chemiluminescence (ECL) detection kit (Amersham Biosciences, Chalfont St. Giles, UK). The primary antibodies used were Phospho-Akt, Akt, Phospho-Erk, Erk1/2, Phospho-JNK, JNK, Phospho-p38, p38, and GAPDH.

### Statistical analysis

All data are presented as the mean ± standard deviation (SD), and statistical analyses were performed using the statistical software Statistical Package for the Social Sciences (SPSS) version 19.0. Student’s *t* test was used to analyze the difference between the control and conditioned medium-cultured groups. *P* < 0.05 was considered statistically significant.

## Results

### Characterization of ADSCs

**Fig. 1:**
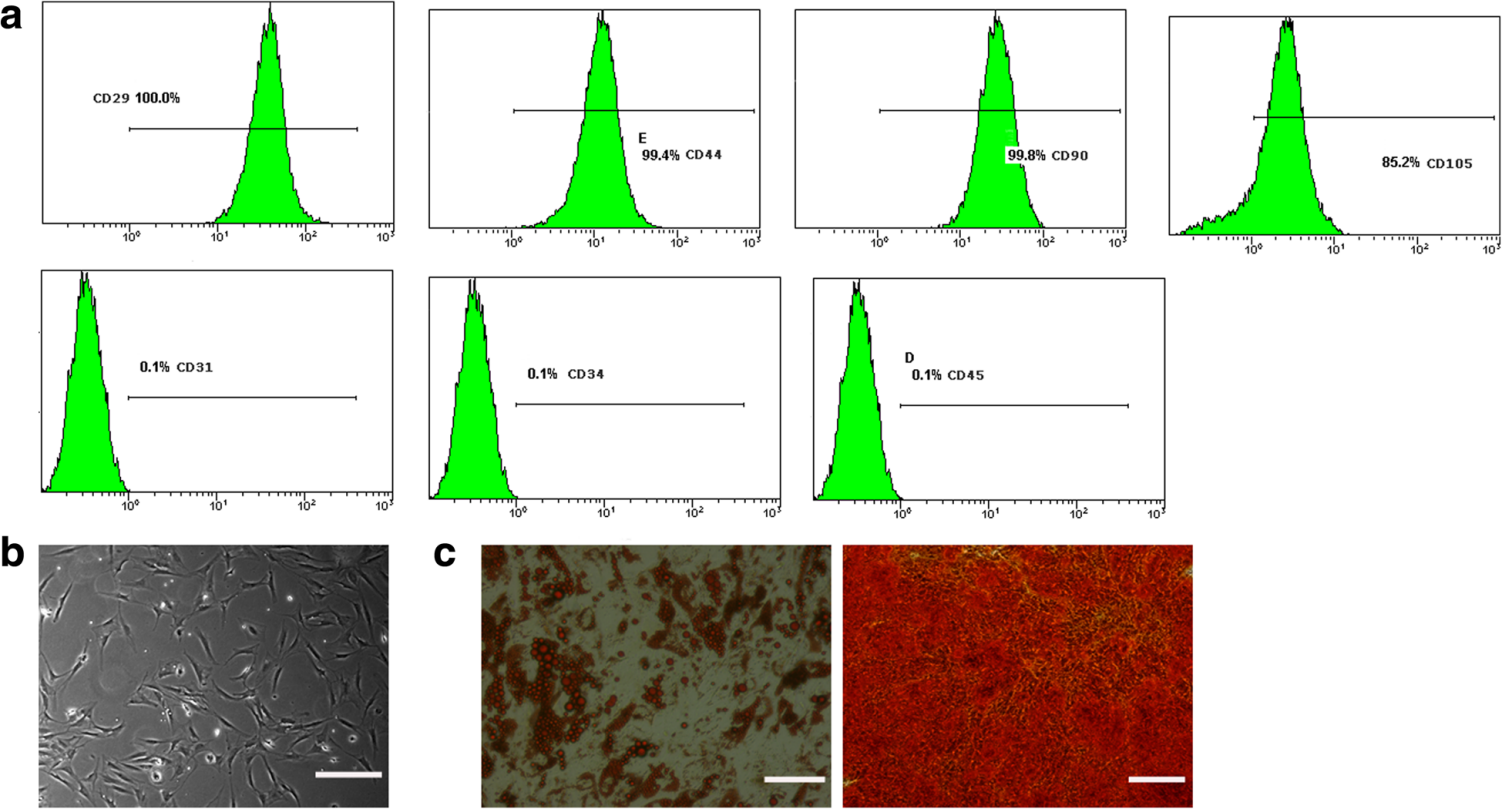
Characterization of human adipose-derived stem cells (ADSCs). **a** Flow cytometric characterization of ADSCs. ADSCs strongly expressed CD29, CD44, CD90, and CD105, and did not express CD31, CD34, or CD45. **b** ADSCs exhibited a fibroblast-like morphology. **c** Cells were induced to differentiate into adipocytes (left panel) and osteoblasts (right panel); scale bars = 100 μm

As reported previously [13], human ADSCs displayed positive staining for the specific mesenchymal stem cell surface markers CD29 (100%), CD44 (99.4%), CD105 (85.2%), and CD90 (99.8%), and negative staining for the hematopoietic stem cell surface markers CD31 (0.1%), CD34 (0.1%), and CD45 (0.1%) (Fig. 1a). Throughout the whole incubation period, human ADSCs exhibited a typical fibroblast-like morphology (Fig. 1b). We examined the multipotential differentiation capacity of ADSCs using adipogenic and osteogenic assays. ADSCs were induced with adipogenic medium for 2 weeks and developed an adipogenic phenotype, as shown by Oil Red O staining (Fig. 1c). We also cultured ADSCs with osteogenic medium for 3 weeks and stained them with Alizarin Red S to confirm mineralization; staining indicated the presence of calcium deposits (Fig. 1c). The results demonstrated that the isolated ADSCs showed typical ADSC characteristics.

### ADSC-CM reduced the ECM-related gene expression in KFs

**Fig. 2:**
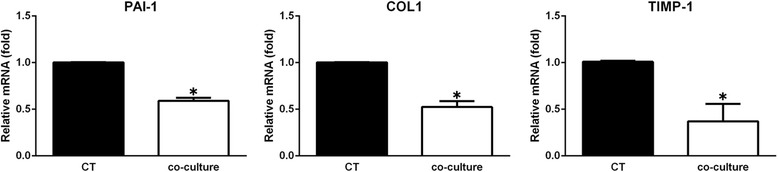
ADSC-CM inhibits the gene expression of plasminogen activator inhibitor-1 (PAI-1), collagen I (COL I), and tissue inhibitor of metalloproteinase 1 (TIMP1) in keloid fibroblasts. qPCR was employed to analyze the expression of various genes in the cells that were cultured with or without ADSC-CM for 5 days. The gene expression levels are presented relative to the control (CT) level (*P < 0.05)

Plasminogen activator inhibitor (PAI)-1 has been shown to play important roles in the progression of tissue fibrosis, and collagen accumulation can be attenuated by inhibiting PAI-1 in keloids [14]. After coculture for 5 days, PAI-1 gene expression dropped to 59.12% of the control group. Increasing deposition of ECM is characterized by overexpression of COL1 and COL3, but COL1 gene expression decreased by 47.6% (Fig. 2). However, there was no significant difference in COL3 expression between the two groups. Tissue inhibitor of metalloproteinase (TIMP)1 is a glycoprotein of the TIMP family. Preliminary experiments revealed that TIMP1 may result in deposition of COL1 in keloids [15]. In our study, we found that TIMP1 decreases significantly in keloids after coculture with human ADSC-CM. All the differences between the control group and the experimental group proved significant.

### ADSC-CM inhibited cell proliferation

**Fig. 3:**
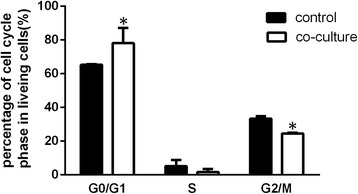
ADSC-CM inhibits keloid fibroblast proliferation. The effect of ADSC-CM on the cell cycle profiles was evaluated by flow cytometric analysis. *P < 0.05, versus control

After culturing keloids with conditioned medium for 24 h, cells in the G0/G1 phase increased and cells in the G2/M phase decreased significantly compared with the control group (Fig. 3).

### ADSC-CM eliminated cell invasion

**Fig. 4:**
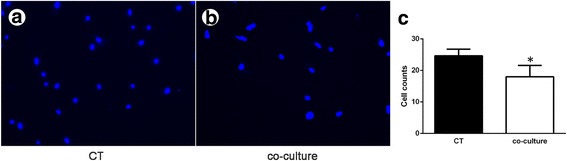
ADSC-CM depresses the invasive abilities of keloid fibroblasts. **a**, **b** As determined by transwell® assay, the migratory keloid fibroblasts were visualized by imaging the nuclei labeled with DAPI. **c** The number of migrated cells was counted in three randomly selected fields. *P < 0.05, versus control (CT)

Because the enhanced invasive properties of dermal fibroblasts are the critical parameters in the development of keloid disease [2], we wondered whether coculture would affect the cell behavior of KFs in vitro. We found that ADSC-CM strikingly reduced the number of cells that migrated across the filter membrane to the lower surface (Fig. 4a, b). Compared with the control group, the experimental group exhibited a noticeable reduction in the number of migrated cells to approximately 25% (Fig. 4c).

### ADSC-CM depleted CD31^+^/CD34^+^ vessels and reduced collagen deposition

**Fig. 5:**
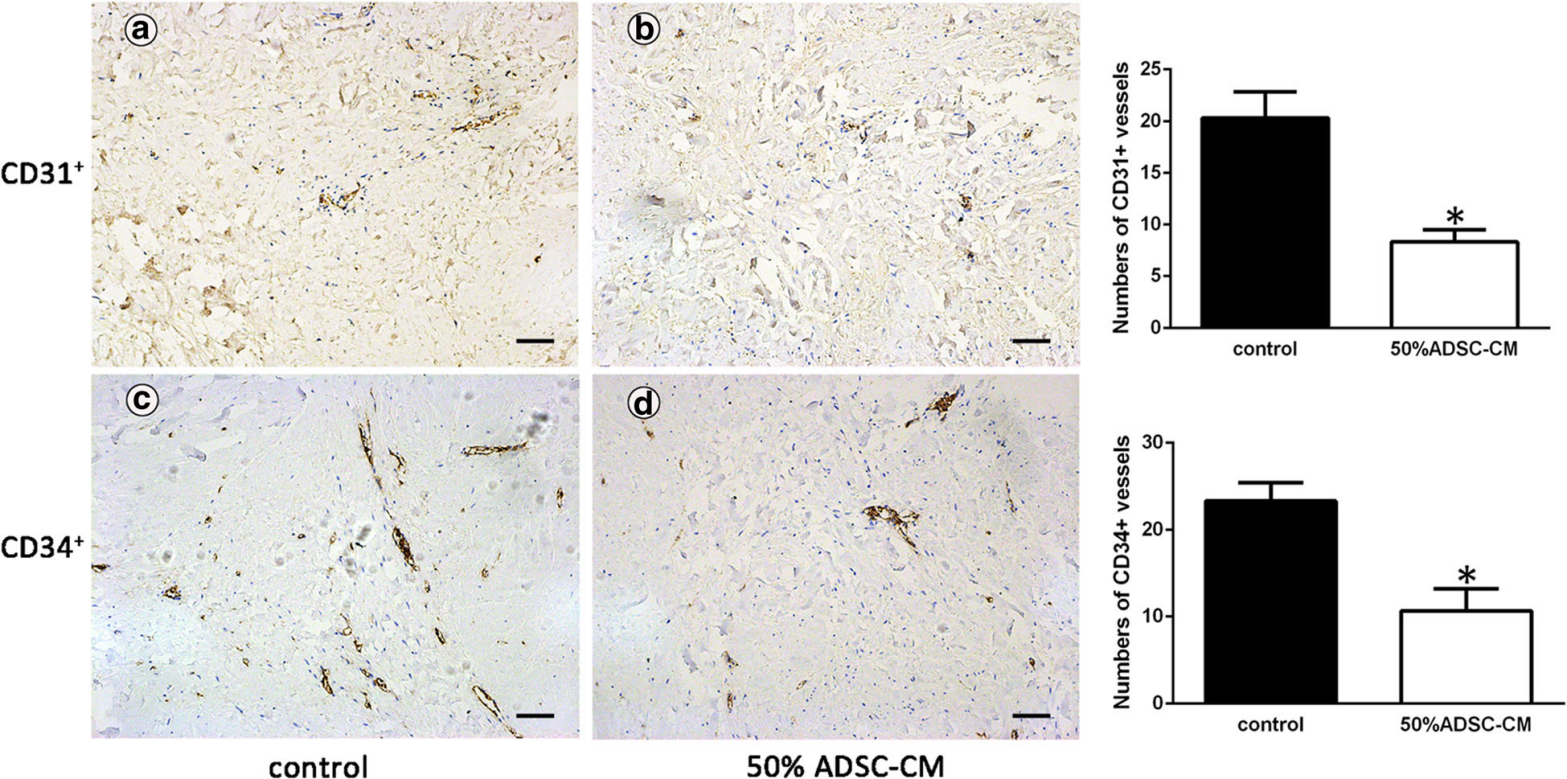
Adipose-derived stem cell-conditioned medium (ADSC-CM) reduces angiogenesis in keloid explant culture. After culturing with (**b**, **d**) or without (**a**, **c**) ADSC-CM for 8 days, the keloid explants were sectioned and subjected to immunohistochemical analysis using antibodies against CD31 (**a**, **b**) and CD34 (**c**, **d**). Brown coloration indicates positive staining of related markers. The numbers of CD31-positive endothelial cells and CD34-positive microvascular endothelial cells were counted in three randomly selected fields for each sample under a microscope. Scale bars = 100 μm. *P < 0.05, versus control

**Fig. 6:**
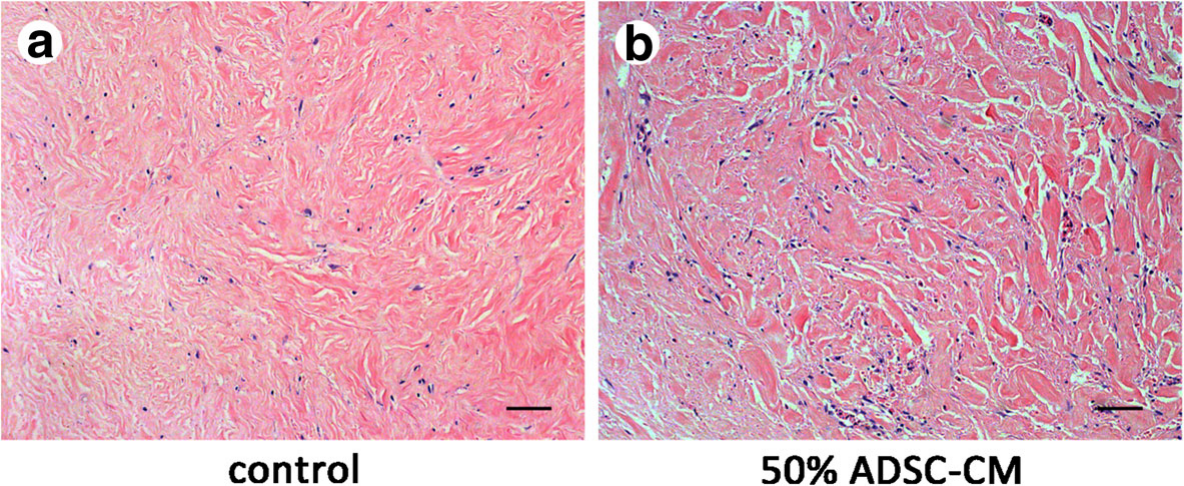
After culturing with (**b**) or without (**a**) adipose-derived stem cell-conditioned medium (ADSC-CM), sections were also subjected to H&E staining for histological analysis. Scale bars = 100 μm

Compared with normal dermis or normal scars, keloids contain an increased vessel density [5]. Hence, we wanted to know whether ADSC-CM could reduce angiogenic development. As expected, we observed significant decreases in the numbers of CD31^+^ and CD34^+^ vessels in the ADSC-CM group (Fig. 5). H&E results showed that collagen had been remodeled and reduced in the experimental group (Fig. 6).

### ADSC-CM reduced protein expression in KFs

**Fig. 7:**
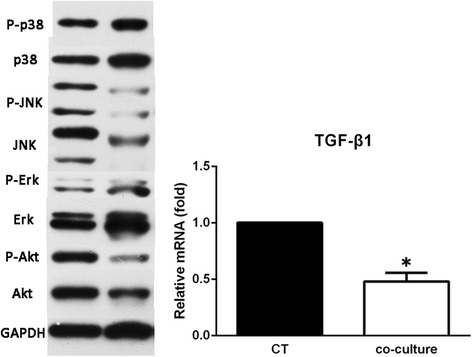
ADSC-CM interferes with intracellular signaling in vitro. To explore the underlying mechanism of the antifibrotic effect of ADSC-CM, the protein level was measured by Western blotting in primary keloid fibroblasts (left panel). Gene expression of transforming growth factor (TGF)-β in the experimental and control (CT) groups (right histogram). *P < 0.05, versus control

To further clarify the intracellular signaling pathways, the activation status of these signaling molecules was examined. We observed that the phosphorylated levels of Akt, ERK1/2, and JNK all decreased moderately after culture with ADSC-CM. However, there was no significant influence on the activation of p38 MAPK, as shown in Fig. 7.

## Discussion

The pathobiology of keloid formation is not completely understood. Some hypotheses have been proposed for the etiology of keloids. As reported in the literature, keloids exhibit ethnic differences in occurrence and frequently occurs in those of Afro-Caribbean or south Indian origin which suggests a genetic factor in keloid etiology [16, 17]. However, there are no reports of any linkage genes in the incidence of keloid disease so far. At the same time, studies have found that keloid development is complex, without specific factors being identified. All these factors have caused difficulties in finding an effective way to treat keloids.

In addition to some traditional therapies, there are many new therapeutic approaches, such as stem cell therapy, that have brought new hope for the management of keloids. Recent studies have shown that MSCs can regulate the wound-healing process and prevent scar formation in keloid development, suggesting that they may have a promising therapeutic role in keloids. The paracrine role of MSCs is also attracting particular attention for a therapeutic effect on wound healing, tissue repair, and scar remodeling [18].

Compared with other MSCs, ADSCs are rich in resources and have the same characteristics as MSCs. Furthermore, studies show that ADSCs facilitate wound healing, inhibit hypertrophic scar fibroblast proliferation, and attenuate wound inflammation. Taken together, this evidence suggests that ADSCs may play a great role in inhibiting keloid fibroblast proliferation and ECM formation. Our team planned to explore this puzzle in this work.

Human ADSCs exhibit fibroblastic and spindle-shaped morphology, and co-express several mesenchymal markers such as CD29, CD44, and CD90. As in a previous study [19], we confirmed that ADSCs could also transdifferentiate into adipocytes and osteoblasts, demonstrating their potential multilineage differentiation abilities. ADSC-derived paracrine molecules have therapeutic potential in keloids.

As mentioned above, the etiology of keloid formation is complex and involves many participating factors. TGF-β1 is recognized as the main factor in promoting keloid formation to date. Furthermore, ECM deposition is a characteristic pathological feature of keloids. Recent reports show that human ADSCs could ameliorate TGF-β1-induced fibrotic changes by significantly decreasing submucosal fibrosis and COL1 expression [20]. In our study, ADSC-CM not only significantly decreased gene expression of TGF-β1, but also attenuated ECM-related COL1 gene expression in keloid fibroblasts. In an in vitro experiment, the structure of collagen was found to become thinner and more orderly after culture with ADSC-CM. Taken together, the data from in vitro and in vivo studies show that ADSC-CM plays a part in reducing ECM formation of keloids especially with respect to COL1.

However, previous studies have reported that ADSC-CM can promote fibrosis [21, 22], which is in contrast to our results. The reason for this may be attributed, to some extent, to the growth conditions of ADSCs and fibroblasts, and different concentrations of ADSC-CM in the mixed medium. ADSC-CM promotes fibroblast proliferation and fibrosis only under normal conditions and inhibits the process under profibrotic conditions, which can explain this discrepancy. Besides, keloid fibroblasts are different from normal fibroblasts with regard to gene expression and growth pattern.

TIMP1 is a glycoprotein of the TIMP family [23]. TIMP1 is one of the important regulators of ECM degradation and remodeling. Studies show that small interfering (si)RNA knockdown of TIMP1 in keloid fibroblasts leads to degradation of COL1 [24]. In our study, ADSC-CM significantly reduced gene expression of TIMP1 by 63.05 ± 10.87%. PAI-1, also known as endothelial plasminogen activator inhibitor, is the principal physiological inhibitor of the plasminogen activator/plasmin protease system [25]. Increased PAI-1 activity has been noted in tissue fibrosis, especially in keloid tissue where PAI-1 was dramatically increased [26]. Emerging evidence demonstrates that the relationship between PAI-1 and collagen accumulation is an important mechanism in the development of keloids [27, 28]. In our experiments, PAI-1 gene expression was inhibited by ADSC-CM, showing a decrease of 40.88 ± 1.77%. Reduced PAI-1 and TIMP-1 gene expression may well show that ADSC-CM can reduce ECM deposition by keloids by regulating collagen degradation and associated gene expression.

Keloid fibroblasts are characterized by a high proliferation capacity and more aggressive properties [5]. As our results show, experimental group fibroblasts were significantly inhibited at the G1 stage of the cell cycle, which implies that ADSC-CM could attenuate keloid fibroblast proliferation. As shown in Fig. 2, keloid fibroblasts in the control group migrated across the bottom of the chamber more than in the experimental group, and the difference between the two groups was statistically significant.

Histologically, keloids contain an increased blood vessel density compared with normal dermis or normal scars [29]. To better mimic the in vivo response to ADSC-CM, human keloid explants were embedded and sectioned for further analyses. As expected, we observed reduced cellularity and microvasculature in ADSC-CM-treated tissue sections. Quantification data revealed that approximately 55% of CD31^+^ cells were reduced by ADSC-CM. Meanwhile, microvascular endothelial cells noticeably declined to 57% in the papillary and reticular dermis, as visualized by CD34^+^ immunostaining.

Recently, scientists have paid more attention to the function of MSCs in inhibiting fibrosis. Similarly, we anticipated that ADSC-CM would contain more factors which could have the potential to inhibit keloid growth. Our results have proved that ADSC-CM can inhibit keloid fibroblast proliferation and reduce ECM-related gene and protein expression.

In summary, we have shown that the blockade of TGF-β/Smad and MAPK/ERK signaling pathways by ADSC-CM can effectively inhibit the proliferation, migration, cellular invasion, and ECM accumulation of KFs and concomitantly reduce the expression of inflammatory cytokines and inhibit angiogenesis, suggesting that ADSC-CM may be an attractive therapeutic intervention in the clinical setting of keloid disorder.

## Conclusions

Our research revealed that ADSC-CM downregulated the extracellular matrix-related gene expression of PAI-1, TIMP-1, and COL1 and inhibited the expression of cell proliferation proteins. Consequently, ADSC-CM attenuated the bioactivities of KFs, providing novel treatment strategies for keloids.

## Abbreviations

ADSC: Adipose-derived stem cell
CM: Conditioned medium
COL1: Collagen 1
COL3: Collagen 3
DAB: 3,3′-Diaminobenzidine
DMEM: Dulbecco’s modified Eagle’s medium
ECL: Enhanced chemiluminescence
ECM: Extracellular matrix
FBS: Fetal bovine serum
H&E: Hematoxylin and eosin
IGF: Insulin-like growth factor
KF: Keloid fibroblast
MSC: Mesenchymal stromal cell
PAI: Plasminogen activator inhibitor
PBS: Phosphate-buffered saline
qRT-PCR: Quantitative real-time polymerase chain reaction
SD: Standard deviation
TGF: Transforming growth factor
TIMP: Tissue inhibitor of metalloproteinase
VEGF: Vascular endothelial growth factor

## References

[1] Philandrianos C, Kerfant N, Jaloux C (2016). Keloid scars (part I): clinical presentation, epidemiology, histology and pathogenesis. Ann Chir Plast Esthet.

[2] Ogawa R. Keloid and hypertrophic scars are the result of chronic inflammation in the reticular dermis. Int J Mol Sci. 2017;18(3). 10.3390/ijms18030606 PMC5372622 28287424

[3] Chalmers RL (2011). The evidence for the role of transforming growth factor-beta in the formation of abnormal scarring. Int Wound J.

[4] Unahabhokha T, Sucontphunt A, Nimmannit U (2015). Molecular signalings in keloid disease and current therapeutic approaches from natural based compounds. Pharm Biol.

[5] Le AD, Zhang Q, Wu Y (2004). Elevated vascular endothelial growth factor in keloids: relevance to tissue fibrosis. Cells Tissues Organs.

[6] Friedenstein AJ, Chailakhjan RK, Lalykina KS (1970). The development of fibroblast colonies in monolayer cultures of guinea pig bone marrow and spleen cells. Cell Tissue Kinet.

[7] Pittenger MF, Mackay AM, Beck SC (1999). Multilineage potential of adult human mesenchymal stem cells. Science.

[8] Puissant B, Barreau C, Bourin P, et al. Immunomodulatory effect of human adipose tissue-derived adult stem cells: comparison with bone marrow mesenchymal stem cells. Br J Haematol 2005;129(1):118–129. 10.1111/j.1365-2141.2005.05409.x 15801964

[9] Cui L, Yin S, Liu W (2007). Expanded adipose-derived stem cells suppress mixed lymphocyte reaction by secretion of prostaglandin E2. Tissue Eng.

[10] Wang X, Gao Z, Wu X (2016). Inhibitory effect of TGF-β peptide antagonist on the fibrotic phenotype of human hypertrophic scar fibroblasts. Pharm Biol.

[11] Wang W, Qu M, Xu L (2016). Sorafenib exerts an anti-keloid activity by antagonizing TGF-β/Smad and MAPK/ERK signaling pathways. J Mol Med (Berl).

[12] Peach M, Marsh N, Macphee DJ (2012). Protein solubilization: attend to the choice of lysis buffer. Methods Mol Biol.

[13] Huang SJ, Fu RH, Shyu WC (2013). Adipose-derived stem cells: isolation, characterization, and differentiation potential. Cell Transplant.

[14] Tuan TL, Wu H, Huang EY, et al. Increased plasminogen activator inhibitor-1 in keloid fibroblasts may account for their elevated collagen accumulation in fibrin gel cultures. Am J Pathol. 2003;162(5):1579–89. 10.1016/S0002-9440(10)64292-7 PMC1851185 12707042

[15] Dong J, Ma Q (2017). TIMP1 promotes multi-walled carbon nanotube-induced lung fibrosis by stimulating fibroblast activation and proliferation. Nanotoxicology.

[16] Ud-Din S, Bayat A (2014). New insights on keloids, hypertrophic scars, and striae. Dermatol Clin.

[17] Bran GM, Goessler UR, Hormann K, Riedel F, Sadick H (2009). Keloids: current concepts of pathogenesis (review). Int J Mol Med.

[18] Jiao Y, Wang X, Zhang J (2017). Inhibiting function of human fetal dermal mesenchymal stem cells on bioactivities of keloid fibroblasts. Stem Cell Res Ther.

[19] Daher SR, Johnstone BH, Phinney DG, March KL (2008). Adipose stromal/stem cells: basic and translational advances: the IFATS collection. Stem Cells.

[20] Sangkum P, Yafi FA, Kim H (2016). Effect of adipose tissue-derived stem cell injection in a rat model of urethral fibrosis. Can Urol Assoc J.

[21] Lee SH, Jin SY, Song JS, Seo KK, Cho KH (2012). Paracrine effects of adipose-derived stem cells on keratinocytes and dermal fibroblasts. Ann Dermatol.

[22] Kim WS, Park BS, Sung JH, Yang JM, Park SB, Kwak SJ (2007). Wound healing effect of adipose-derived stem cells: a critical role of secretory factors on human dermal fibroblasts. J Dermatol Sci.

[23] Stetler-Stevenson WG (2008). Tissue inhibitors of metalloproteinases in cell signaling: metalloproteinase-independent biological activities. Sci Signal.

[24] Aoki M, Miyake K, Ogawa R (2014). siRNA knockdown of tissue inhibitor of metalloproteinase-1 in keloid fibroblasts leads to degradation of collagen type I. J Invest Dermatol.

[25] Cale JM, Lawrence DA (2007). Structure-function relationships of plasminogen activator inhibitor-1 and its potential as a therapeutic agent. Curr Drug Targets.

[26] Tuan TLZJ, Sun B, Nichter LS (1996). Elevated levels of plasminogen activator inhibitor-1 may account for the altered fibrinolysis by keloid fibroblasts. J Invest Dermatol.

[27] Tuan TL, Hwu P, Ho W (2008). Adenoviral overexpression and small interfering RNA suppression demonstrate that plasminogen activator inhibitor-1 produces elevated collagen accumulation in normal and keloid fibroblasts. Am J Pathol.

[28] Syed F, Bagabir RA, Paus R, Bayat A (2013). Ex vivo evaluation of antifibrotic compounds in skin scarring: EGCG and silencing of PAI-1 independently inhibit growth and induce keloid shrinkage. Lab Invest Aug.

[29] Ehrlich HP, Desmouliere A, Diegelmann RF (1994). Morphological and immunochemical differences between keloid and hypertrophic scar. Am J Pathol.

